# Veterinary-led interventions and owner perceptions of male cat sterilization: Insights from a community-based initiative in Malang, Indonesia

**DOI:** 10.14202/vetworld.2025.2113-2126

**Published:** 2025-07-30

**Authors:** Albiruni Haryo, Handayu Untari

**Affiliations:** Department of Veterinary Pathology Anatomy, Faculty of Veterinary Medicine, Brawijaya University, Malang, Indonesia

**Keywords:** community-based intervention, compliance behavior, cultural beliefs, male cat sterilization, One Health, urban animal control, veterinary outreach

## Abstract

**Background and Aim:**

Male cat sterilization is often neglected in feline population control strategies due to cultural beliefs, logistical barriers, and misconceptions, especially in developing urban areas. This study aimed to evaluate the effectiveness of a veterinary-led, community-based intervention in Malang, Indonesia, to promote male cat sterilization, improve owner compliance, and explore sociocultural influences on participation.

**Materials and Methods:**

A descriptive cross-sectional study was conducted among owners of non-pedigree male cats (6 months to 5 years old) in the Greater Malang area. Data were collected through a validated online questionnaire asse-ssing demographic characteristics, sterilization readiness, compliance behavior, and sociocultural perceptions. Quantitative data were analyzed using descriptive statistics and logistic regression, while open-ended responses underwent thematic analysis.

**Results:**

Out of 182 potential respondents, 99 met the inclusion criteria and proceeded with the intervention. A 99% compliance rate was achieved (98/99), supported by strong community engagement and clear procedural guidelines. Education level, previous pet ownership, and affiliation with animal welfare organizations had a significant influence on compliance (p < 0.05). Barriers for non-compliant individuals included scheduling conflicts, financial constraints, and surgical apprehension. Thematic analysis revealed that cultural beliefs about masculinity and religious ambiguity were common deterrents, but community outreach and peer influence effectively shifted perceptions.

**Conclusion:**

This study demonstrates that structured, culturally attuned, veterinary-led community interventions can achieve high compliance in male cat sterilization. Strategic partnerships with local organizations, transparent communication, and logistical support are key to success. Findings support the integration of such programs into broader One Health initia-tives, emphasizing responsible pet ownership, zoonotic disease mitigation, and sustainable management of urban animal populations.

## INTRODUCTION

Cat overpopulation is a growing concern in urban areas worldwide, particularly in developing regions where large populations of free-roaming cats pre-sent significant public health, environmental, and ani-mal welfare challenges. In such settings, population control efforts have largely prioritized spaying female cats, given their well-established role in preventing unwanted litters [1, 2]. However, the role of male cats in perpetuating uncontrolled breeding remains insufficiently addressed and demands greater focus [3]. The global disparity in sterilization rates between male and female cats highlights a widespread issue that transcends regional boundaries.

Research consistently associates higher rates of female spaying with factors such as the urgency to prevent pregnancy and the accessibility of public health programs aimed at reducing kitten overpo-pulation [1, 2]. In contrast, male cats, despite their capacity to impregnate multiple females within short periods, are often overlooked in sterilization initiatives. This imbalance is exacerbated by practical obstacles, including financial limitations and restricted access to veterinary services [3]. Cultural and social norms further complicate efforts to neuter male cats. In various communities, prevailing notions of masculinity and beliefs in the natural vitality of male cats deter owners from choosing sterilization [4–6]. These cul-turally ingrained attitudes, often tied to broader views on reproduction and gender roles, contribute to wide-spread resistance. The failure to neuter male cats carries broader implications, including increased public health risks from zoonotic diseases and environmental issues associated with unchecked breeding [7, 8].

Despite global recognition of the need for feline population control, current efforts disproportionately emphasize the spaying of female cats, with minimal attention given to the role of male cats in perpetuating unplanned breeding cycles [1–3]. This imbalance is evident across multiple regions and persists even in urban centers with active sterilization programs. Alth-ough male cats can impregnate numerous females in short timeframes, they are frequently excluded from targeted campaigns due to societal perceptions, financial constraints, and limited public education on their reproductive impact [3–6]. Moreover, few studies have systematically examined how cultural beliefs, socioeconomic factors, and logistical barriers influence owner decisions regarding male cat sterilization. Partic-ularly in Southeast Asia, and Indonesia in particular, empirical evidence on community-driven approaches to promoting neutering remains sparse. Existing literature often overlooks male-focused interventions and lacks in-depth exploration of how local norms and community networks can be leveraged to enhance compliance and uptake. This gap underscores the need for localized research that not only assesses behavioral readiness but also integrates veterinary leadership and culturally sensitive outreach to address barriers specific to male cat sterilization.

This study aimed to evaluate the effectiveness of a veterinary-led, community-based intervention in promoting male cat sterilization in Malang, Indonesia. Specifically, the research sought to: (1) Assess owner readiness and compliance with sterilization protocols; (2) identify demographic, socioeconomic, and cultural factors associated with sterilization decisions; and (3) explore the role of local animal welfare organizations in facilitating behavior change. By combining quantita-tive measures of compliance with qualitative insights into owner perceptions, this study provides a comprehensive understanding of the enablers and obstacles influencing male cat sterilization uptake. The findings aim to inform scalable strategies for urban animal population control, improve public health through zoonotic disease prevention, and contribute to the broader One Health framework by addressing the interconnectedness of human, animal, and environmental health.

## MATERIALS AND METHODS

### Ethical approval

The study protocol was independently reviewed and approved by the Ethics Committee of Satwa Sehat Veterinary Hospital (Document No. EC/RSH-SS/VM/001/2025). The committee confirmed that all proposed procedures comply with veterinary-medical standards and the hospital’s Standard Operating Pro-cedures for ethical research. All surgical or clinical interventions were performed by licensed veterina-rians. Study data were coded to protect owner confidentiality. The committee determined that the protocol adequately safeguards animal welfare and owner privacy and therefore granted full ethical approval.

Potential respondents received an information sheet detailing the study’s objectives, risks, and bene-fits, and were reminded of their right to withdraw at any point without negative consequences. Digital consent was documented before questionnaire completion to ensure awareness of data use and privacy protocols. All personally identifiable data were securely stored and handled in accordance with applicable data protection regulations to maintain confidentiality throughout the study.

### Study period and location

**Figure 1:**
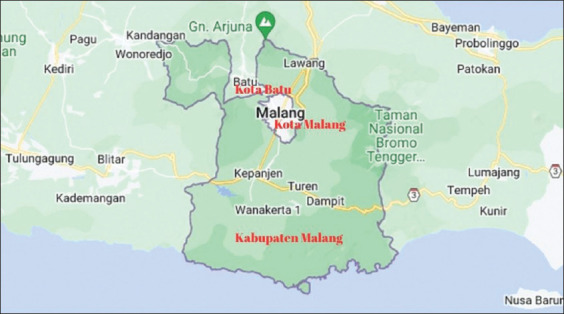
Map of research locations in Malang, East Java, Indonesia (Source: Badan Informasi Geospasial (BIG) [9]).

The study was conducted during July and August 2024 in Malang, East Java, Indonesia – an urban region facing growing challenges in controlling the cat population (Figure 1) [9]. This location was selected due to its active network of animal welfare communities and documented issues concerning free-roaming male cats, making it an ideal site for examining behavioral responses to targeted sterilization efforts.

### Study design

This study employed a descriptive cross-sectional design to evaluate owner readiness and compliance with a community-based sterilization initiative. Such designs are commonly used in veterinary public health to assess behaviors and attitudes at a single point in time, without requiring longitudinal follow-up [10, 11].

Participant readiness and compliance were dete-rmined by adherence to scheduled appointment times, meeting the health-related requirements on the surgical date, ensuring the cat fulfilled the eligibility criteria (i.e., healthy, aged 6 months to 5 years), and implementing both pre- and post-operative procedural guidelines.

### Participant eligibility and sampling strategy

The study targeted adult cat owners residing in the Greater Malang area, which includes Kota Malang, Kabupaten Malang, and Kota Batu (Table 1). Initial outreach through local community networks and social media groups yielded 182 potential respondents, ali-gning with recommended practices for inclusive sam-pling in community-based research [12]. The figure of 182 potential respondents comprised the entire roster of eligible adult owners of non-pedigree male cats identified across Greater Malang through systematic mapping of local animal-welfare organisations and social-media groups, consistent with the inclusive purp-osive-sampling principles that guide community-based research. Of these, 99 respondents provided usable data for analysis.

**Table 1:** Distribution of final respondents by region.

Region
Kota Malang
Kabupaten Malang
Kota Batu

After eligibility confirmation, which required ownership of at least one non-pedigree male cat aged 6 months to 5 years and willingness to participate in the sterilization campaign, a total of 99 individuals were confirmed for final data analysis.

Eligibility criteria for non-pedigree male cats aged 6 months to 5 years are generalizable because they help reduce not only health risks associated with breeding, but also behavioral issues observed in intact males. The most common problematic behaviors include aggres-sion toward people or other animals, inappropriate elimination, and fear-based responses [13]. A study by Oliveira-Martins *et al*. [14] found that sterilization performed before 4 months of age or at/after 6 months yields similar health outcomes in both canine and feline species.

Furthermore, sterilization is biologically rele-vant for controlling non-pedigree cat populations, preventing overpopulation, and mitigating associated risks of disease transmission and behavioral concerns (e.g., territorial aggression and marking). Male cats can be neutered from 6 months of age due to earlier physical maturity and reduced susceptibility to joint issues. Sterilization in male animals has also been shown to reduce the risk of testicular cancer and prostatic hyperplasia [15].

A purposive sampling method was used to recruit cat owners affiliated with animal welfare organizations or social media groups. This strategy enhances repre-sentativeness in behavior-focused research by targeting individuals with pre-existing interest or awareness of pet health [11]. Key demographic information, including age, residence, and the number of cats owned, was recorded to support later analyses of factors influencing compliance.

### Questionnaire development and validation

The questionnaire was administered in Bahasa Indonesia. It was developed and validated in acco-rdance with established best practices for community-based veterinary research, as outlined by Becker *et al*. [16] and Lavan *et al*. [17].

Initial drafts were based on frameworks from existing surveys on pet owner attitudes and sterilization campaigns, ensuring content relevance and reducing the need to build a new instrument from scratch [16].

The instrument was refined through expert consu-ltation and a pilot test involving 15 cat owners not included in the main sample. This process evaluated clarity, cultural relevance, and question appropriate-ness, following guidelines for pre-validation and piloting [17, 18].

To account for variability in literacy and digital fluency within the target population, the final questio-nnaire was designed to feature simplified language and clear instructions. Sections were organized to mini-mize respondent fatigue, aligning with Mwacalimba *et al*.’s [19] recommendation to keep surveys concise and user-friendly.

### Data collection procedure

Data collection was performed through an online questionnaire. Both closed- and open-ended questions were included, supporting a mixed-methods approach to assess compliance and explore motivations and concerns surrounding sterilization [20].

The questionnaire gathered information on owner demographics, cat health, prior awareness of sterilization benefits, and logistical factors such as transportation methods, thereby providing a comprehensive profile of compliance determinants [16, 17].

To reduce potential bias from self-reporting, the questionnaire was structured to encourage honesty and clarity in responses. Open-ended questions were specifically included to capture nuanced views and perceptions not easily addressed by closed-ended items. This was particularly important for understanding motivations behind decision-making in settings where cultural myths and economic factors might shape attit-udes toward sterilization.

Sampling for this study used a purposive method, allowing for the selection of respondents who met specific inclusion criteria (owners of male cats aged 6 months to 5 years).

The sample was drawn from urban and semi-urban areas in Malang, ensuring a diverse representation of pet owners across various socioeconomic backgrounds. A total of 182 potential respondents were initially identified, of whom 99 were ultimately included in the final study based on their willingness and eligibility.

The sampling process aimed to balance a range of demographic factors, including socioeconomic status, prior pet ownership, and cultural background, to capture a wide spectrum of attitudes and behaviors regarding sterilization. This design enabled analysis of how variables such as education level, income, and pet ownership experience might influence decisions about male cat sterilization.

### Quantitative data analysis

Quantitative and qualitative data were analyzed separately using a mixed-methods framework that integrates numerical indicators of compliance with thematic insights into owner attitudes [12].

Descriptive statistics were used to summarize frequencies and proportions, particularly in relation to readiness and adherence to health and transportation guidelines for sterilization. These analyses provided an initial overview of compliance distribution.

Subsequently, Chi-square tests and logistic regression were used to examine relationships betw-een compliance and demographic variables [17]. All statistical analyses were conducted using SPSS version 26 (IBM Corp., NY, USA), with a significance threshold set at p < 0.05. Odds ratios were computed to measure the strength of association between predictors and compliance outcomes.

Missing data were addressed using multiple imputations through SPSS’s built-in function, which improves accuracy by estimating values based on existing patterns in the dataset. This method was sele-cted to reduce bias caused by incomplete responses.

For cases where missing values remained after imputation, sensitivity analysis was conducted to deter-mine their potential influence on final results.

### Qualitative data analysis

Open-ended responses were subjected to the- matic analysis to uncover recurring themes and owner pers-pectives on sterilization practices [20]. Two researchers (AH and HU) independently coded the transcripts using NVivo version 12 (QSR International Pty Ltd, MA, USA), followed by a collaborative review and reconciliation of categories to enhance inter-rater reliability.

This approach ensured that diverse viewpoints were considered and mitigated potential interpretation bias. The analysis provided richer insights into owner motivations, cultural beliefs, and perceived obstacles - factors that could not be captured through quantitative measures alone.

By integrating statistical and thematic findings, the study gained a more comprehensive understanding of how community-led interventions influence behavioral readiness and sterilization compliance.

### Reliability and validity measures

Throughout the instrument development and data analysis phases, specific steps were taken to ensure the reliability and validity of the results. In line with Becker *et al*. [16] and Lavan *et al*. [17], these steps included pilot testing, expert review, and iterative refinement of questions for clarity and relevance.

To promote consistent interpretation, explanatory notes and a standardized introduction were embedded in the online questionnaire.

In addition, incomplete or contradictory responses were flagged and subjected to follow-up review, reducing the likelihood of erroneous data inclusion [18].

The methodological rigor employed supports transparency and replicability, in accordance with accepted practices in community-based veterinary research [12, 19].

By integrating validated instruments, incorporating ethical safeguards, and employing a mixed-methods approach, this study presents a comprehensive meth-odology for understanding the multifaceted factors influencing male cat sterilization.

The detailed procedures presented here serve as a practical model for future interventions that aim to leverage community engagement to enhance parti-cipation in veterinary public health programs.

## RESULTS

### Participant demographics

**Figure 2:**
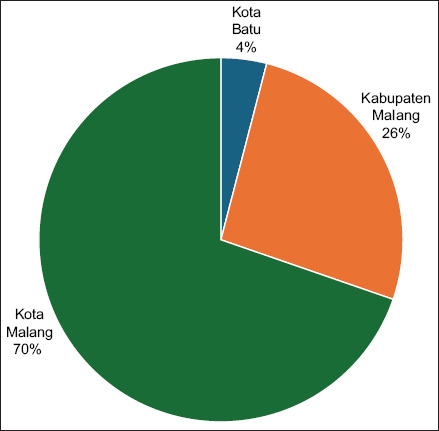
Respondent distribution by region.

Ninety-nine respondents met the inclusion criteria, each owning at least one non-pedigree male cat aged 6 months to 5 years. The largest proportion (n = 69) resided in Kota Malang, followed by Kabupaten Malang (n = 26) and Kota Batu (n = 4) (Figure 2).

This distribution aligns with previous findings indicating that urban residents are generally more engaged in veterinary health initiatives due to their proximity to animal welfare services and heigh-tened exposure to stray cat populations. While urban concentration suggests greater awareness of steriliz-ation programs, it also highlights disparities in outreach between urban and semi-urban areas [21, 22].

Within the total sample, the mean age of the respondents was 35.2 years (standard deviation ± 7.1), suggesting that a relatively mature demographic was likely to have had prior exposure to community-based animal welfare campaigns. In terms of gender distribution, female respondents constituted 58.6% (n = 58), while male respondents accounted for 41.4% (n = 41). A small fraction of respondents (approximately 3%) reported being first-time pet owners, whereas the majority had previously owned cats or other pets concurrently, suggesting a baseline familiarity with veterinary care routines. This observation is consi-stent with the literature that suggests that prior pet ownership fosters greater openness to sterilization initiatives [23, 24].

To further assess the relationship between demographic factors and compliance, respondents were asked to provide details on household composition, including the presence of children or other dependents. Approximately 42% of respondents (n = 42) lived in households with children under the age of 12. While a direct analysis between parental status and sterilization intent was not performed at this stage, preliminary cross-tabulations suggested that households without children reported a slightly higher inclination to follow through on sterilization appointments, reflecting exist-ing studies that link fewer family distractions with more consistent pet care [23, 24].

A suggestion for presenting these demographic attributes in detail would be a tabular format, enabling readers to compare relevant factors such as age, gender, parental status, and prior pet ownership. These data points will inform subsequent analyses on how demographic subgroups engage with sterilization programs. A tabular summary (Table 2) of respondents demographics highlight key variables that may affect readiness and compliance in a male cat sterilization campaign.

**Table 2:** Demographic profile of the study respondents (n = 99).

Demographic variable	Respondent
Age (Mean ± SD)	SD ± 7.1
Gender (Female: Male)	Female 58.6% (n = 58): Male 41.4% (n = 41)
Location	Kota Malang (n = 69) Kabupaten Malang (n = 26) Kota Batu (n = 4)
Households with children	42% Respondents
First-time pet owners: A study of the benefits	3% Respondents
Previous cat ownership experience	97% Respondents

SD=Standard deviation

### Socioeconomic characteristics and educational attainment

In addition to basic demographic details, the respondents were asked about their educational attainment and approximate household income lev-els. Educational attainment was classified into four categories: Secondary school or below (n = 15), high school diploma (n = 35), undergraduate degree (n = 38), and postgraduate degree (n = 11). Although not a formal prerequisite for participation, this stratification allowed the study to explore correlations between education levels and awareness or attitudes toward sterilization. Results indicated that respondents with undergraduate or postgraduate degrees collectively displayed a higher self-reported understanding of the benefits of sterilization, including population con-trol, health advantages, and mitigation of nuisance behaviors, than those with high school or lower-level qualifications. These findings are consistent with a broader literature that links higher education levels to a greater understanding of veterinary practices [25, 26].

A parallel pattern emerged concerning household income, broadly grouped into three brackets: Low (less than Indonesian Rupiah [IDR] 3 million/month; n = 23), middle (IDR 3–6 million/month; n = 52), and high (above IDR 6 million/month; n = 24). Most respondents fell within the middle-income bracket, reflecting the typical wage distribution in urban Indonesian settings. Interestingly, respondents in the high-income bracket were more likely to articulate confidence in being able to afford additional post-operative care, such as specialized cat carriers, follow-up veterinary visits, and premium diets for recovery. These revelations align with prior research suggesting that financial capacity is a critical determinant of compliance, particularly when unforeseen complications or subsequent treatments are involved [21, 22].

### Affiliation with animal welfare organizations

**Figure 3:**
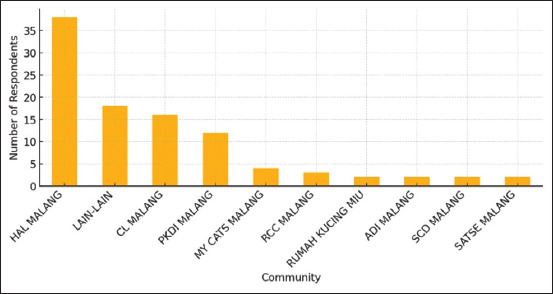
Participation by animal welfare community.

In line with the integral role of local animal welfare organizations, this study examined the relationships between community-based affiliations and respondent engagement. Among the 99 respondents, affiliations spanned 13 registered or informal animal welfare gro-ups, including HAL MALANG, Rumah Kucing MIU, PKDI Malang, and several smaller independent collectives. HAL MALANG accounted for the largest proportion of affiliated respondents (n = 45), followed by Rumah Kucing MIU (n = 25) and PKDI Malang (n = 12). The remaining respondents (n = 17) were distributed across smaller groups (Figure 3).

Participants were asked to evaluate how organizational involvement influenced their decision to neuter male cats. Of the respondents, 76% (n = 75) indicated that access to veterinary resources, inform-ational sessions, and peer endorsements “strongly influenced” their compliance. This attests to the potency of grassroots advocacy in dispelling myths, addressing concerns, and facilitating logistical arrang-ements [22, 27]. Numerous respondents identified personal conversations with group leaders or peers as key motivators for participation. These narratives align with previous observations that owner-to-owner testim-onials can substantially reshape perceptions of risk and challenge misconceptions surrounding sterili-zation [24, 28].

The synergy between animal welfare organizations and the veterinary-led initiative also manifested in the provision of low-cost services, transport coordination, and follow-up care guidelines. Outreach materials circulated through social media and community gathe-rings effectively disseminated essential information on benefits, potential risks, and post-operative proced-ures. Such a comprehensive approach corroborates research highlighting that sustained educational out-reach can significantly improve participation rates, especially when local organizations are directly inv-olved [27, 28]. Consequently, the success observed in this study highlights the need for continued collaboration, underscoring how these networks can serve as conduits for both technical guidance and social support.

### Cultural and religious beliefs

The open-ended responses examined the respondents’ beliefs, cultural perspectives, and religious considerations that may influence their decision-making process regarding male cat sterilization. Appr-oximately 28% of the respondents (n = 28) reported initially hesitating to neuter their male cats due to misconceptions, such as the notion that sterilization would alter a cat’s personality or diminish its natural instincts [25, 29]. A smaller subset (n = 12) mentioned encountering familial or peer opposition rooted in cultural ideals of male virility, echoing narratives from other regions where male cats are perceived as symbolic guardians or representations of masculinity [30].

Religious beliefs also play a nuanced role. While Islam is the predominant faith in the region, none of the respondents explicitly cited doctrinal prohibitions against sterilization. Instead, concerns revolve around a general uncertainty about whether sterilization might conflict with ethical or spiritual frameworks that promote compassion toward animals. In this regard, the following points were raised: The respondents emphasized the need for guidance from religious authorities or respected community leaders [29, 30]. It is pertinent to note that local religious leaders have, in certain cases, publicly endorsed sterilization as a humane approach to controlling stray populations and preventing suffering among neglected animals. This stance appears to have alleviated apprehensions among devout owners, suggesting that interventions incorporating religious or cultural validation could effec-tively enhance participation.

Overall, the data revealed that while cultural and religious factors can create hesitancy, direct engagement through educational sessions, community discussion forums, and one-on-one consultations effectively disp-elled many misconceptions [26]. Participants who reported initial reluctance frequently transitioned to stronger support for sterilization after receiving thorough explanations about its health benefits and alignment with responsible animal stewardship. Hence, a key takeaway involves the strategic use of culturally sensitive communication to dismantle lingering myths and ensure that pet owners perceive sterilization as both ethically and socially acceptable [22, 24].

### Compliance with sterilization protocols

A central objective of this study was to evaluate respondent adherence to key stipulations, including ensuring the cat met the eligibility criteria (i.e., healthy, aged 6 months to 5 years), following quarantine protocols before the procedure, transporting the cat in lockable carriers, and respecting scheduled appointment times. Results indicated a strikingly high compliance rate, with 98 of 99 respondents fully adhering to the core requirements. Only one respondent failed to meet the health criteria on the scheduled day, citing an unexpected bout of respiratory infection in the cat. This result reflects a high degree of respondent preparedness and planning, particularly when compared with the modest compliance levels often reported in broader literature on pet sterilization campaigns [31, 32].

Several factors likely contributed to this favorable outcome. First, respondents received detailed instruct-ions and reminders through multiple channels (email, text, and social media group announcements), mini-mizing confusion about procedural guidelines. Second, most respondents had ties to the local animal welfare organizations mentioned earlier, suggesting that peer pressure and social accountability may have heightened adherence [27, 28]. Finally, the program’s transparent structure outlining benefits, risks, and post-operative care appears to have fostered trust and alleviated anxiety, further reinforcing owner commitment to following through on the requirements [26].

In terms of behavioral readiness, respondents gene-rally expressed optimism about their ability to handle pre- and post-operative responsibilities, including administering medication, ensuring adequate rest, and monitoring for complications. Follow-up inquiries revealed that 95% (n = 94) of those who completed the procedure reported no significant post-surgical com-plications. The remaining 5% mentioned minor issues, such as delayed wound healing or transient appetite loss, but they adhered to the veterinarian’s instructions for resolution. Overall, these data indicate the efficacy of clear communication, supportive comm-unity networks, and accessible veterinary guidance in achieving near-complete compliance for male cat sterilization.

### Dropouts and barriers to participation

Although compliance levels were generally high, the initial pool of 182 potential respondents was narrowed to 99 final respondents. An analysis of the 83 respondents who did not complete the program offers insight into the broader challenges that sterilization initiatives face. In particular, 35 of these individuals cited scheduling conflicts, as their work hours or family commitments clashed with the allocated time for the sterilization service. Another 20 reported difficulties securing appropriate transport, expressing concerns about long-distance travel and the stress it might place on their cats [27, 33]. Some prospective respondents (n = 12) noted a sudden change in financial conditions, such as job loss or unexpected expenses, which rend-ered them reluctant to incur even nominal costs assoc-iated with the procedure [34].

Apprehension about surgical risks contributed notably to respondent attrition. Specifically, ten individuals expressed apprehension about anesthesia, potential complications, or insufficient post-operative care, aligning with prior studies that cited perceived surgical risks as a deterrent to sterilization [31, 32]. Furthermore, six of the unaccounted individuals did not respond to follow-up messages, making it difficult to ascertain their exact reasons for disengagement. Nonetheless, these results underscore that logistical, financial, and psychological barriers can collectively undermine campaigns that otherwise present a clear scientific or welfare rationale [22].

The single respondent who attended but was ultimately non-compliant did so due to the last-minute discovery of an upper respiratory tract infection in the cat, which contraindicated immediate surgery. While this scenario was unique, it highlights the importance of pre-procedure health checks and contingency plans, such as rescheduling or offering alternative appointments. To visualize the main reasons for attrition, a bar chart could be used to illustrate the frequency of each category relative to the total. This graphical representation provides a clear overview of recurring hurdles and can inform more adaptive planning in future interventions.

### Summary of key observations

In synthesizing the outcomes of this study, several salient patterns emerged. First, demographic and socioeconomic indicators encompassing factors such as educational level, household income, and prior experience in pet ownership correlated with heightened readiness to sterilize male cats, echoing broader scholarship on compliance dynamics [21, 22]. Participants with more financial resources or advanced education were more apt to understand and accept sterilization benefits, although targeted support mec-hanisms (e.g., subsidized fees) effectively broadened participation among mid- to lower-income groups.

Second, local animal welfare organizations played an indispensable role in disseminating information and facilitating logistical arrangements, with many respondents emphasizing the motivational power of communal endorsement [27, 28]. This network-based approach was instrumental in dispelling myths and alleviating fears associated with religious or cultural misgivings. While certain cultural notions surrounding male cat virility and “natural” behavior persisted, direct engagement, coupled with comprehensive educational content, succeeded in reshaping most respondents’ perceptions [29, 30].

Third, the campaign’s structured guidelines, bolstered by consistent communication, contributed to an exceptionally high compliance rate among those who remained in the program, with 98 out of 99 respondents meeting all procedural requirements. This outcome illustrates that meticulously designed interventions encompassing clear scheduling, readiness assessments, and follow-up support can effectively counteract the logistical and psychological hurdles that often undermine sterilization initiatives [32].

In summary, the results of this study provide a detailed portrait of how demographic profiles, socio-economic status, organizational support, and cultural narratives interplay to shape male cat sterilization outcomes in an urban Indonesian context. The near-total compliance rate among final respondents stands as evidence that carefully orchestrated campaigns, reinforced by local animal welfare organizations and culturally attuned messaging, can significantly enhance sterilization uptake [24]. However, the attrition noted in the larger group indicates that more inclusive strategies, potentially involving alternative scheduling options, stronger financial backing, or clearer reassurances regarding surgical safety, could further broaden participation. These findings lay a critical foundation for the subsequent discussion, in which the implications for future policy design, community-based veterinary interventions, and sustainable population control effo-rts are examined.

## DISCUSSION

### Effectiveness of the community-based intervention

This study highlights the effectiveness of a veterinary-led community-based intervention for achieving exceptionally high compliance rates in male cat sterilization. In contrast to prior reports of moderate adherence, the near-total compliance in this study illustrates the potential of integrated outreach and community engagement [35, 36]. This section contextualizes the results within broader scholarship, addresses alternative interpretations, and explores practical implications. This review further discusses methodological strengths, research limitations, and directions for future inquiries.

### Interpreting high compliance rates

A central outcome in this study was the remarkable level of compliance among the final parti-cipants, with only one respondent failing to fulfill the health-related requirements on the appointed surgical date. Such uniform adherence might initially appear atypical; however, it aligns with existing models in which strong community engagement and provision of low-cost services can yield elevated uptake [35, 37]. A considerable number of lower- to middle-income respondents also expressed readiness to comply with sterilization, provided that costs remained subsidized or manageable. This highlights the potential efficacy of targeted financial assistance programs, such as sliding-scale fees or sponsorships by animal welfare organizations, which can enable a broader demographic to participate in sterilization initiatives. Thus, although socioeconomic status influences compliance levels, it does not necessarily inhibit participation when cost-mitigation measures are in place [27, 28].

In our case, respondents not only received direct messaging through social media and community networks but also relied on trusted peers and com-munity leaders for logistical and emotional support. The combination of community support and professio-nal guidance effectively mitigated common barriers, including financial constraints, misinformation about surgery, and fear of complications, thereby enabling owners to comply with veterinarian-recommended protocols.

Historically, community-led interventions that emphasize social support and education have been associated with high adherence to sterilization initi-atives [33]. Our findings support this notion, revealing that local animal welfare organizations play a crucial role in shaping perceptions, clarifying the procedure’s rationale, and providing tangible support mechanisms, such as transport coordination. In many cases, group affiliation was cited as a decisive factor in overcoming cultural and logistical obstacles, suggesting that harn-essing collective solidarity and shared accountability within pet owner groups can be a decisive catalyst for participation [24, 25].

### Alternative explanations for compliance

As with all interventions reporting strong parti-cipation, the possibility of social desirability bias should be considered. Participants may have overstated their willingness or downplayed concerns in self-reported responses that aimed to align with perceived communal norms [38].

In addition, a self-selection effect may have occurred: Individuals already favoring sterilization, motivated by previous positive experiences or heigh-tened welfare awareness, would naturally be more inclined to enroll and complete the program. This phenomenon could inflate observed compliance rates because neutral or skeptical owners might have ex- cluded themselves earlier in the recruitment process [39]. The study relies on volunteers or individuals who are already involved in animal welfare networks. These respondents, who are often more engaged in the community and have greater prior knowledge of animal welfare practices, may naturally exhibit higher compliance rates than the general population.

However, several mitigating factors suggest that such biases only partially explain the high compliance rate. First, the dropouts from the initial pool of 182 potential respondents included those who lacked the resources or confidence to proceed. This drop-off indicates that the study sample is not entirely composed of respondents predisposed to compliance, as some individuals who may not have had the financial or logi-stical means to proceed expressed an initial interest. The inclusion of these individuals in the dropout analysis helps address the concern of self-selection bias, as it highlights that the high compliance rate is not solely attributable to a preselected group of respondents but rather reflects a broader spectrum of the population.

Second, the embedded verification methods, which require owners to demonstrate evidence of health checks and adhere to transport conditions, imply that actual compliance, not merely stated intent, was measured. Reliance on objective measures rather than solely on self-reported compliance minimizes the likelihood that respondents overstate their commit-ment. The use of health checks and transportation conditions serves as an additional safeguard against social desira-bility bias, as respondents were required to demonstrate concrete actions rather than merely declare their intentions. However, it is essential to note that although these methods enhance the reliability of the findings, they may not entirely eliminate the potential for bias if the verification process itself is susceptible to inconsistencies or errors in documentation.

Despite these measures, the potential for bias remains, especially regarding the self-reporting of attitudes and behaviors. Future studies that aim to eliminate social desirability bias entirely could incor-porate longitudinal follow-ups, respondent obser-vation, and on-site confirmations during sterilization events [40].

### Community engagement models and their efficacy

The success of male cat sterilization initiatives often hinges on collaborative frameworks that tie academic or veterinary expertise to local community structures [41]. Various published models illustrate the value of partnerships that unify diverse stakeholders. For instance, the Community Research Liaison Model facilitates aligned goals between researchers and the local populace, while pop-up or mobile clinics operated by veterinary institutions lower logistical hurdles [36, 42]. In this study, participation was bolstered by outreach to 13 animal welfare organizations, effectively bridging any communication gaps between veterinarians and cat owners. These organizations disseminated vital information and provided peer-based reassurance to hesitant owners, neutralizing misconceptions about surgery outcomes and cultural taboos.

Furthermore, integrated approaches that involve human social services can broaden the impact’s scope, particularly in low- to middle-income areas. Linking free or subsidized sterilization drives to ancillary ser-vices, such as nutrition counseling and immunization campaigns, can enhance overall engagement and embed sterilization within a more comprehensive fram-ework of community health [35, 42]. Although this study did not implement such multi-faceted outreach, the high compliance rate suggests ample scope for synergy if future initiatives incorporate additional supp-ort measures, thereby reaching even those with limited awareness or resources.

### Sociocultural and economic dynamics

The demographic analysis revealed that education level, prior pet ownership experience, and income bracket were correlated with a stronger willingness to comply with male cat sterilization. These findings align with broader scholarship, suggesting that higher socioeconomic status is often correlated with greater engagement in responsible pet ownership [21, 22]. However, the sizable middle-income group in this study, which comprised nearly the same rate, challenges the assumption that only affluent demographics will participate. Cost-reduction strategies, such as subsidized fees, alleviated financial burdens for lower-income respondents, demonstrating that monetary assistance can reduce barriers and expand participation [36].

Cultural myths surrounding male cats’ “natural state” and perceptions of virility were addressed thr-ough targeted education. While some owners initially hesitated due to these beliefs, consistent messaging and endorsement from recognized community figures reshaped attitudes, fostering acceptance of sterilization as both humane and beneficial. This highlights the importance of culturally sensitive communication strategies in influencing compliance [27, 29, 30].

### Methodological considerations

Adopting a mixed-methods research approach, which included both quantitative metrics (e.g., com-pliance rates, demographic correlations) and qualitative insights (e.g., open-ended survey responses, anecdotal feedback), yielded a rich perspective on respondents’ experiences and program efficacy. This mixed-methods approach addresses common methodological limitations in animal welfare research by providing a triangulated dataset [40]. The validated survey instrument further bolstered credibility, enabling consistent measurem-ent of key variables, such as health readiness, transport compliance, and cultural beliefs, across a diverse sample [43].

Nonetheless, certain limitations remain. First, the study employed a purposive sampling method that prioritized existing community networks, potentially biasing the sample toward individuals predisposed to engage in or trust animal welfare activities. Second, the study was conducted over a short duration, assessing only immediate compliance without longitudinal mon-itoring of cat health outcomes or changes in community norms [44]. A longitudinal design, possibly integrating re-checks or respondent observation, could better capture the durability of attitudinal shifts and behavioral adherence [39]. Finally, the differential dropout between initial recruitment and final participation signals that barriers such as schedule conflicts, transport challenges, and fear of surgery remain unresolved for a considerable subgroup [22]. Accounting for these factors in the design of future campaigns, such as offering more flexible time slots or enhanced post-operative support, could further enlarge the respondent base.

### Building on Trap-Neuter-Return (TNR) and community outreach models

This intervention builds on established TNR and community outreach models by integrating more structured and proactive community support systems. Unlike traditional TNR models, which often rely on volunteers and sporadic community engagement, our approach involves continuous collaboration with local animal welfare organizations and the active involvement of community leaders. These leaders played a critical role in reshaping cultural perceptions about sterilization, overcoming resistance based on myths surrounding male cat virility and the natural state of cats. Furthermore, this initiative expanded the scope of typical TNR models by actively addressing the logistical and financial barriers that many pet owners face, such as transport difficulties and affordability issues, which have been identified as significant barriers to participation in other sterilization campaigns [36, 42].

While previous studies have shown the benefits of community-based engagement in TNR programs, our model explicitly targets both cultural beliefs and socioeconomic factors, thereby enhancing the inclusivity and sustainability of the intervention. By integrating zooanthropological insights and emphasizing community-driven advocacy, this model provides a more comprehensive framework for feline population control, strengthening the One Health approach by addressing both the health of humans and animals, as well as the environmental impact of uncontrolled feral cat populations.

### Scalability in resource-limited and rural settings

A strength of this intervention is its potential for scalability, especially in resource-limited or rural settings. Although the present study focused on urban and semi-urban areas, the principles of this intervention can be adapted to different contexts. However, scalability in rural areas may face additional challenges, including limited access to veterinary services, fewer community-based organizations, and logistical difficulties in transportation. In resource-poor regions, the cost of sterilization can also be a significant barrier, necessitating the integration of financial support models, such as subsidized fees or partnerships with non-governmental organizations.

To address these challenges, future initiatives could utilize mobile veterinary clinics or collaborative outreach models that involve local community organizations. This would reduce logistical barriers, such as the transportation of animals, and enhance access to veterinary care in rural areas. By leveraging existing community networks and building local partnerships, the intervention can be adapted to operate with lower resources while maintaining high compliance rates.

Moreover, integrating local community leaders and trusted peers in these areas is crucial to overcoming cultural resistance and ensuring the long-term sustainability of sterilization efforts. Similar to urban areas, the active involvement of community-based organizations and religious or cultural leaders can help reshape attitudes toward sterilization, making the intervention more acceptable and effective.

### Practical implications

The demonstrated efficacy of an organized, community-based approach offers valuable insi-ghts for policymakers, veterinarians, and nonprofit organizations aiming to curb cat overpopulation. By channeling resources through existing local networks, these stakeholders can leverage trust, familiarity, and shared accountability to encourage responsible pet ownership [44]. Specifically, creating low-cost steri-lization initiatives and coupling them with educational workshops or digital campaigns can not only raise awareness but also sustain compliance over extended periods. Lessons from TNR models, which have shown enduring success in stabilizing feral cat populations [35], may also be integrated by targeting semi-owned and community cats.

In addition, the robust adherence demonstrated underscores the efficacy of transparent guidelines and stepwise instructions. Rather than overwhelming owners with technical jargon, a user-friendly outline covering pre-operative preparedness, transport protocols, and post-operative care can alleviate apprehension. When combined with direct support such as providing carriers, scheduling follow-ups, or offering help lines for emergency queries, these structured interventions create a positive feedback loop that instills confidence and normalizes sterilization [27].

The findings of this study also have significant implications for national and regional policy in Indonesia, where rapid urbanization and stray animal populations are growing concerns. The success of this community-driven sterilization program suggests that integrating low-cost sterilization initiatives into national and regional public health frameworks could effectively reduce the population of stray cats, addressing not only animal welfare issues but also the public health risks associated with zoonotic diseases. Policymakers should consider scaling up such programs by leveraging local animal welfare organizations and community leaders to promote the One Health approach to public health policy.

Furthermore, the study’s findings support the idea that sterilization programs should be part of broader urban management strategies, including policies on waste management and green space planning, which can support sustainable wildlife control. Given the success of this model, regional governments should explore policy integration at both municipal and pro-vincial levels, ensuring that sterilization campaigns are not isolated interventions but are embedded within comprehensive urban and health policies.

### Study limitations

While these results are promising, they should be interpreted with caution due to the study’s inherent constraints. The reliance on self-reported data raises questions about potential reporting bias, despite measures taken to validate actual compliance [38]. The sample, primarily recruited through animal welfare organizations, may not accurately represent individuals who are either entirely unfamiliar with sterilization or harbor deeply entrenched cultural aversions [30]. In addition, the regional specificity of this intervention, which focuses on urban and peri-urban settings in Malang, limits the external validity of the findings in rural areas, where infrastructure and community structures may differ considerably [44].

Moreover, the lack of a true control group or baseline measurement complicates the capacity to isolate programmatic effects from other concurrent influences, such as broader educational campaigns on social media or seasonal fluctuations in cat breeding behavior. Future research could employ quasi-experi-mental designs or matched comparison groups to improve causal inference [40]. Finally, a longer-term perspective would be beneficial for assessing the sustainability of sterilization’s impact, including whether cat population counts actually decrease and whether community attitudes remain favorable [39].

### Future directions

Given the high levels of compliance observed, a natural next step is to explore the replicability in diverse contexts. Researchers could adapt the current model, emphasizing local partnerships, clear messaging, and logistical support to rural areas or regions where cultural or religious frameworks differ significantly [9, 30]. Multi-site or comparative studies could clarify how demographic, sociocultural, and infrastructural factors influence the uptake of sterilization [37].

In addition, a mixed-methods longitudinal design that tracks feline health outcomes, stray population metrics, and owner attitudes over 1 or 2 years could offer robust evidence of efficacy. Incorporating adva-nced behavioral metrics such as microchipping for re-identification or GPS tracking of neutered cats would provide objective measures of roaming and breeding changes post-sterilization [40]. Triangulating these data with ongoing qualitative assessments would offer a more comprehensive evaluation of community-level change [35].

In summary, the high compliance rates in this study highlight the potential for well-structured, com-munity-centric approaches to substantially improve uptake in male cat sterilization. By combining veterinary expertise with localized advocacy, misconceptions and financial barriers can be overcome, enabling owners to view sterilization as an integral part of responsible pet stewardship. Although social desirability bias remains a possibility, the strong concordance between self-reported and observed behaviors supports genuine engagement with the initiative’s aims. The insights gleaned from the demographic and qualitative data underscore how socioeconomic status, cultural norms, and the presence of supportive community organiz-ations jointly steer owner decisions. Future efforts can build on these findings to develop strategies that foster long-term, inclusive participation, ultimately setting the stage for more coherent and lasting solutions to urban cat overpopulation.

## CONCLUSION

This study demonstrated that a veterinary-led, community-based intervention can achieve exceptionally high compliance rates for male cat sterilization, even in environments where cultural norms or logistical challenges typically hinder partic-ipation. By integrating structured outreach, clear proc-edural instructions, and robust community support, the initiative successfully addressed common barriers, including misconceptions, financial limitations, and access constraints. These findings make a meaningful contribution to the field of feline population manag-ement, highlighting the strategic importance of invol-ving male cats in sterilization campaigns to achieve comprehensive control. The results further underscore the value of combining veterinary expertise with grass-roots networks to enhance transparency, challenge cultural resistance, and strengthen owner commitment to sustainable animal welfare practices.

Importantly, the study exemplifies a One Health approach, illustrating the critical interconnection betw-een human, animal, and environmental health in the context of urban wildlife management. Beyond animal welfare benefits, the initiative also helps reduce the risk of zoonotic disease transmission, such as toxoplasmosis and cat scratch fever, particularly relevant in densely populated areas where unsterilized and free-roaming cats pose significant public health concerns. Promoting male cat sterilization thus serves a dual function: Safeguarding public health while supporting humane population control. Future programs should more exp-licitly embed One Health principles, fostering a holistic understanding of sterilization as a public health measure that contributes to environmental resilience and urban health equity.

The study also highlights the effectiveness of a mixed-methods research design in capturing both quantifiable compliance outcomes and rich, qualitative insights into owner perspectives. This methodological approach enabled a deeper understanding of how demographic, cultural, and socioeconomic variables shape compliance behavior. While high adherence may have been influenced by respondent self-selection and localized community dynamics, the intervention model presented is scalable and adaptable to other urban contexts confronting similar overpopulation challenges.

To maximize replicability and expand impact, future initiatives should incorporate zoonotic risk com-munication and urban environmental management into their core design. This can include education about zoonotic diseases and clearer messaging on the human health benefits of sterilization. Framing feline population control as a zoonotic disease prevention strategy may also enhance engagement across diverse demographic groups. Such framing can increase awareness of the broader health implications of sterilization, fostering deeper and more inclusive community participation.

Moreover, integrating sterilization programs with urban environmental policies, such as waste reduction strategies and green space planning, could further support sustainable population control. For instance, reducing food waste that sustains feral cat populations may enhance sanitation and decrease ecological disruption. Cross-sector collaboration among urban planners, veterinarians, public health professionals, and environmental experts can position sterilization as a core component of a multidisciplinary strategy to improve urban health, biodiversity, and quality of life.

This study not only confirms the value of veteri-nary-led, community-driven sterilization in addressing cat overpopulation but also underscores the urgent need for policy-level integration. Policymakers should recognize the interconnectedness of human, animal, and environmental health as a foundational principle of public health strategy. The findings advocate for the inclusion of One Health frameworks in public policy, urban planning, and public health initiatives focused on disease prevention, sustainable development, and animal welfare in urban areas. Future research should build upon this work through longitudinal studies and cross-cultural comparisons to assess the long-term effectiveness and adaptability of these findings. Maintaining a central focus on One Health will ensure that future interventions meaningfully address the complex challenges of urban wildlife management, zoonotic risk mitigation, and environmental sustainability.

## AUTHORS’ CONTRIBUTIONS

AH: Planned and designed the study, proce-ssed samples, analyzed data, interpreted results, and drafted and revised the manuscript. HU: Study selection, sample monitoring and capture, data analysis, interpreted results, and drafted and reviewed the manuscript. All authors have read and approved the final manuscript.
